# Global analysis of expression profile of members of *DnaJ* gene families involved in capsaicinoids synthesis in pepper (*Capsicum annuum* L)

**DOI:** 10.1186/s12870-020-02476-3

**Published:** 2020-07-09

**Authors:** Fang Fei Fan, Fawan Liu, Xian Yang, Hongjian Wan, Yunyan Kang

**Affiliations:** 1 College of Horticulture, South China Agricultural University, Guangzhou, 510642 PR China; 2 Horticultural Research Institute, Yunnan Academy of Agricultural Science, Kunming, 650231 PR China; 3 State Key Laboratory for Managing Biotic and Chemical Threats to the Quality and Safety of Agro-products, Institute of Vegetables, Zhejiang Academy of Agricultural Sciences, Hangzhou, 310021 PR China; 4 China-Australia Research Centre for Crop Improvement, Zhejiang Academy of Agricultural Sciences, Hangzhou, 310021 China

**Keywords:** DnaJ protein, Pepper, Expression analysis, Phylogenetics, Abiotic stresses

## Abstract

**Background:**

The DnaJ proteins play critical roles in plant development and stress responses. Recently, seventy-six DnaJ genes were identified through a comprehensive bioinformatics analysis in the pepper genome. However, there were no reports on understanding of phylogenetic relationships and diverse expression profile of pepper DnaJ genes to date. Herein, we performed the systemic analysis of the phylogenetic relationships and expression profile of pepper DnaJ genes in different tissues and in response to both abiotic stress and plant hormones.

**Results:**

Phylogenetic analysis showed that all the pepper DnaJ genes were grouped into 7 sub-families (sub-family I, II, III, IV, V, VI and VII) according to sequence homology. The expression of pepper DnaJs in different tissues revealed that about 38% (29/76) of pepper DnaJs were expressed in at least one tissue. The results demonstrate the potentially critical role of DnaJs in pepper growth and development. In addition, to gain insight into the expression difference of pepper DnaJ genes in placenta between pungent and non-pungent, their expression patterns were also analyzed using RNA-seq data and qRT-PCR. Comparison analysis revealed that eight genes presented distinct expression profiles in pungent and non-pungent pepper. The *CaDnaJs* co-expressed with genes involved in capsaicinoids synthesis during placenta development. What is more, our study exposed the fact that these eight DnaJ genes were probably regulated by stress (heat, drought and salt), and were also regulated by plant hormones (ABA, GA3, MeJA and SA).

**Conclusions:**

In summary, these results showed that some DnaJ genes expressed in placenta may be involved in plant response to abiotic stress during biosynthesis of compounds related with pungency. The study provides wide insights to the expression profiles of pepper DanJ genes and contributes to our knowledge about the function of DnaJ genes in pepper.

## Background

The DnaJ protein was originally identified in *Escherichia coli* as a 41-kDa heat shock protein [1]. Generally, it was composed of four domains, including a J-domain, a G/F-domain, a zinc finger (CxxCxGxG) domain, and C-terminal sequences [2–5]. Previous research has attempted to separate DnaJ protein into three groups (I/II/III) according to the characteristics of structure [6]. Group I consisted of the J-domain, G/F-domain, and zinc finger domain. Group II was composed of the J-domain and either a G/F- or a zinc finger- domain. Group III have only the J-domain [7].

Plant growth and development is a complex biological process regulated by a network of various mechanisms. Numerous DnaJ proteins have been reported to participate and play important role in this process. It is well known that photosynthesis which takes place in chloroplast is a vital physiological process to plant growth and development [8]. Previously, researchers have revealed that DnaJ proteins were significant for chloroplast development. For instance, the DnaJ protein ARC6 located in chloroplast membrane of Arabidopsis was functions as a key factor essential for chloroplast differentiation by assembling or stabling the FtsZ rings [9]. Subsequently, Chen et al. [10] found that both specific and cross-talk functions exist in the three small chloroplast-targeted DnaJ proteins, AtJ8, AtJ11 and AtJ20. These small proteins participate in multiple physiological and biochemical processes, including optimization of CO_2_ fixation, in stabilization of PSII complexes and balancing the electron transfer reactions [10]. Recently, members of the DnaJ-like zinc finger domain proteins in *Arabidopsis thaliana* have been reported to participate in the biogenesis and/or the maintenance of plastids. One of the protein family members, PSA2, was found to localize to the thylakoid lumen and regulate the accumulation of photosystem I. The result demonstrated that PSA2 affects chloroplast development [11]. What’s more, considerable research about the function of the DnaJ protein in rice was consistent with that reported previously. Zhu et al. [12] clearly illuminated that the OsDjA7/8 is required for chloroplast development in rice and it may act in relation with other proteins directly or indirectly.

The plant DnaJ functions were not only related to plant growth and development, but also were involved in plant resistance to abiotic stresses. The *BIL2* gene localized in mitochondrial was classified as a member of the DnaJ family [13]. BIL2-overexpression plants showed resistance against salinity stress and strong light stress in *Arabidopisis thaliana* [14]. A chloroplast-targeted *D*na*J* protein (*LeCDJ1*) was isolated from tomato (*Solanum lycopersicum*). The expression was upregulated under chilling stress. Furthermore, the researchers further reported that overexpression of *LeCDJ1* improved tolerance of PSII to low temperature stress, whereas inhibition of *LeCDJ1* enhanced the sensitivity of PSII to low temperature. The result showed that *LeCDJ1* played an important role in maintaining PSII under low temperature stress [15]. In 2015, the second tomato chloroplast-targeted DnaJ protein, *SlCDJ2*, was identified in transgenic tomatoes. The researchers reported that, under heat stress, over-expression of *SlCDJ2* exhibited higher Rubisco activity, Rubisco large subunit content, and CO_2_ assimilation capacity but lower in antisense plants compared with wild-type plants. The results indicated that overexpression of *SlCDJ2* in tomato improved its thermotolerance by alleviating heat stress-induced damage of Rubisco and facilitated, whereas its inhibition enhanced Rubisco damage and reducing heat-induced damage.

Pepper (*Capsicum annum* L*.*), one of important vegetable crops, had been widely cultivated around the world [16]. The pungency of chili fruits, one of the most important ingredients, is due to a group of chemical analogs known as capsaicinoids [17]. Although the biosynthetic pathways (the phenylpropanoid pathway and the branched-chain fatty acid pathway) of capsaicinoid have been reported [18], the factors that regulate these pathways are not clear.

In recent years, the sequencing of whole plant genomes has provided an opportunity to identify different gene families [19–22]. In previous study, we identified a total of 76 putative pepper DnaJ genes (CaDnaJ01 to CaDnaJ76) using bioinformatics methods in the pepper genome [23]. A genome-wide analysis of CaDnaJ gene family was performed to reveal gene structure, conserved motifs, chromosomal localization, *cis*-element and expression profiles in different tissues (root, stem, leaf, and pericarp) and heat stress condition. A number of stress-related *cis*-elements were found in the promoter region of most *CaDnaJ* genes, suggesting that the *CaDnaJs* might be involved in the response process of complex stress conditions [23].

In the current study, to shed light into their underlying relationships, two pepper breeding lines (007EA-pungent and P2-nonpungent) were selected for analyzing co-expression patterns of the factors related capsaicinoid biosynthesis and CaDnaJ genes. Moreover, these CaDnaJ genes response to multiple stresses were also performed using qRT-PCR. These results will facilitate the functional studies of pepper DnaJs in the future.

## Results

### Phylogenetic analysis of pepper CaDnaJ genes

**Fig. 1:**
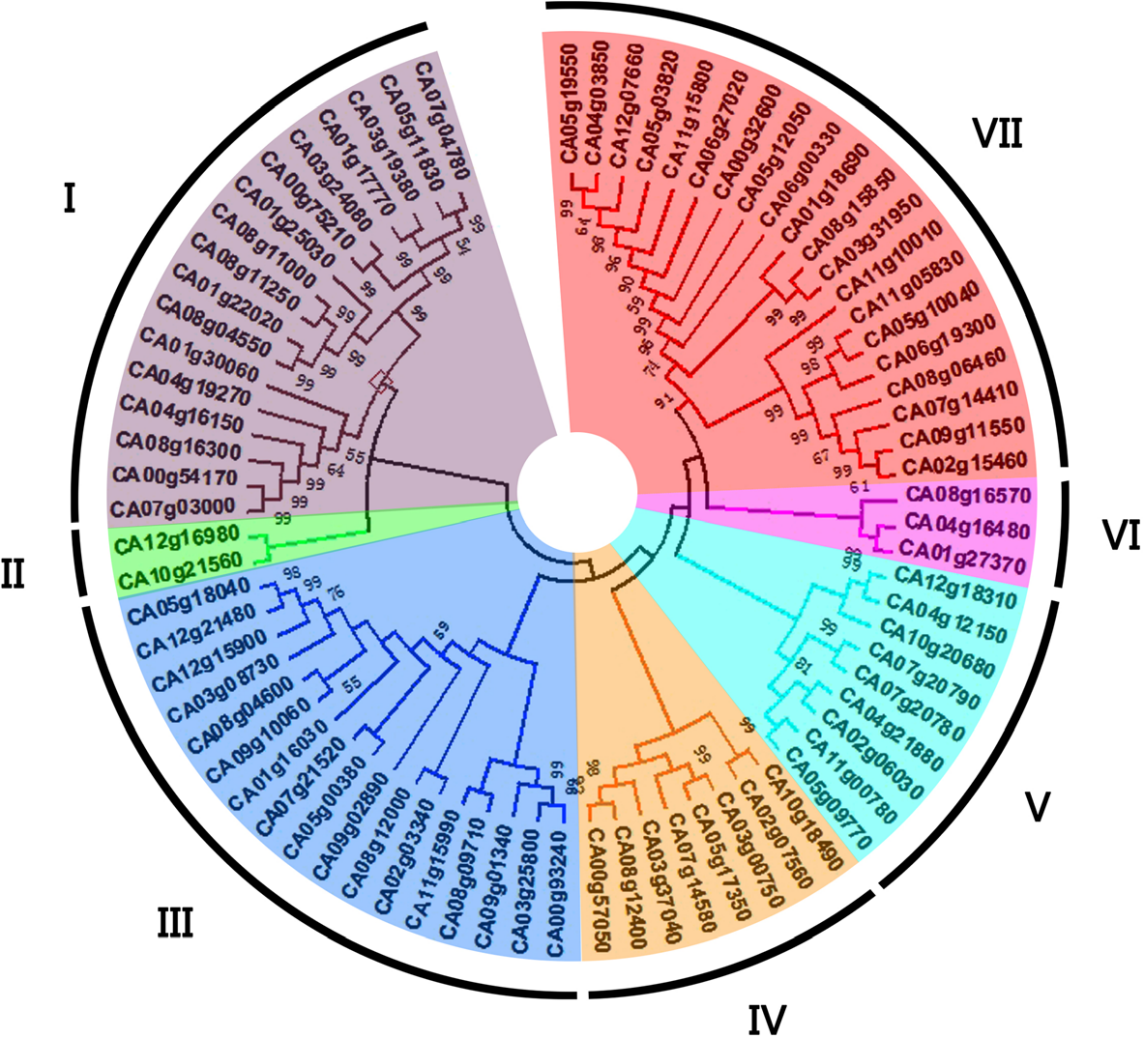
Phylogenetic relationship of the identified 76 CaDnaJ genes in CM334 hot pepper. These genes were divided into seven subfamilies (I, II, III, IV, V, VI and VII)

**Fig. 2:**
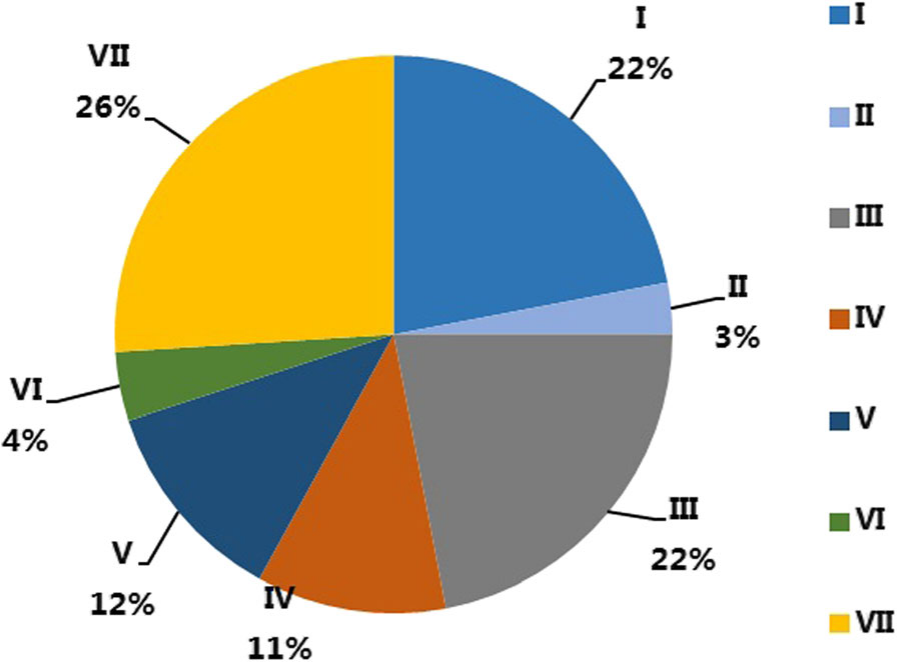
Subfamily constitution of the identified 76 CaDnaJs in CM334

Based on the sequence homology of the 76 DnaJ protein sequences, the pepper DnaJ gene family was grouped into 7 sub-families (sub-family I, II, III, IV, V, VI and VII). As shown in Fig. 1, Sub-family VII (containing 20 members) was the largest group, followed by sub-family I (17), sub-family III (17), sub-family V (9), sub-family IV (8), and sub-family VI (3). The sub-family II was the smallest, with only two *CaDnaJ* gene members (Fig. 2).

### Expression profiles of pepper CaDnaJ genes

Plant tissue/organ-specific expression analysis based on RAN-seq and qRT-PCR technology can provide vital clues about the function differentiation of the genes in different tissue/organs [24–27]. To compare in detail the CaDnaJ expression in CM334, the RNA-seq data of the CaDnaJ genes were selected for further analysis. Different tissues from CM334, including root, stem, leaf, seven stages of pericarp and placenta (6 DPA, 16 DPA, 25 DPA, MG, B, B5, B10, respectively), were selected for expression analysis in the present study.

**Fig. 3:**
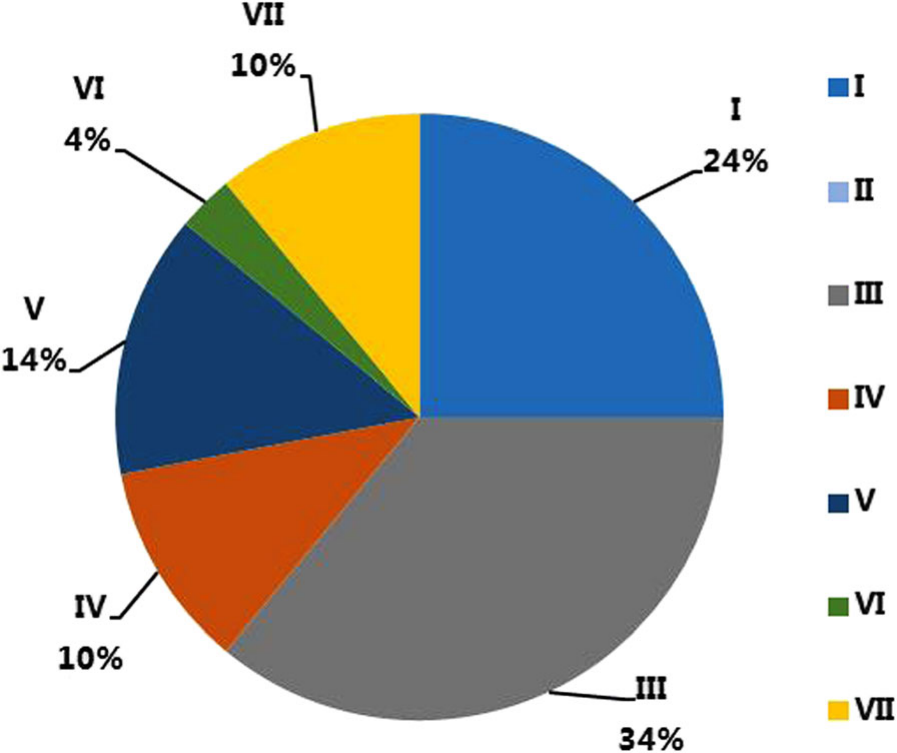
CaDnaJ genes expressed in the investigated plant tissues based on transcriptome data in CM334. RPKM value over 5.0 is defined as expressed genes. 29 CaDnaJs are expressed in at least one tissue. Proportions of CaDnaJ subfamilies are showed in the figure

We found that 29 out of identified 76 CaDnaJ genes were expressed in at least one tissue ([RPKM] ≥ 5.0 was defined as expressed) (Supplement Table 2). The analysis on the constitutions of each subfamily revealed the differential expressions of CaDnaJ subfamilies in tested tissues (Table 1). The subfamily III members possess over 34% of the expressed CaDnaJ in tested tissues (Fig. 3), although it possesses only 22% of the total detected CaDnaJs (Fig. 2), indicating a higher expression level and possible crucial roles in pepper development. In contrast, 26% of detected CaDnaJs belong to the subfamily VII (Fig. 2), while the expressed subfamily VII genes in the tested tissues possess only 10% in all analyzed tissues (Fig. 3). Interestingly, a special situation was found in subfamily II members, the two members were not expressed in the tested tissues (Fig. 3, Supplement Table S2).

**Table 1:** Subfamily constitution of expressed *CaDnaJ* genes in different tissues in pepper

	Leaf	%	Root	%	Stem	%	Pericarp	%	Placenta	%
I	1	0.07	5	0.2	3	0.17	6	0.24	5	0.21
III	8	0.53	10	0.42	8	0.44	9	0.36	9	0.37
IV	0	0	3	0.13	1	0.06	3	0.12	3	0.13
V	3	0.2	3	0.13	3	0.16	3	0.12	3	0.13
VI	1	0.07	1	0.04	1	0.06	1	0.04	1	0.04
VII	2	0.13	2	0.08	2	0.11	3	0.12	3	0.12
Total	15	100	24	100	18	100	25	100	24	100

### Expression patterns of CaDnaJ genes in various tissues

**Fig. 4:**
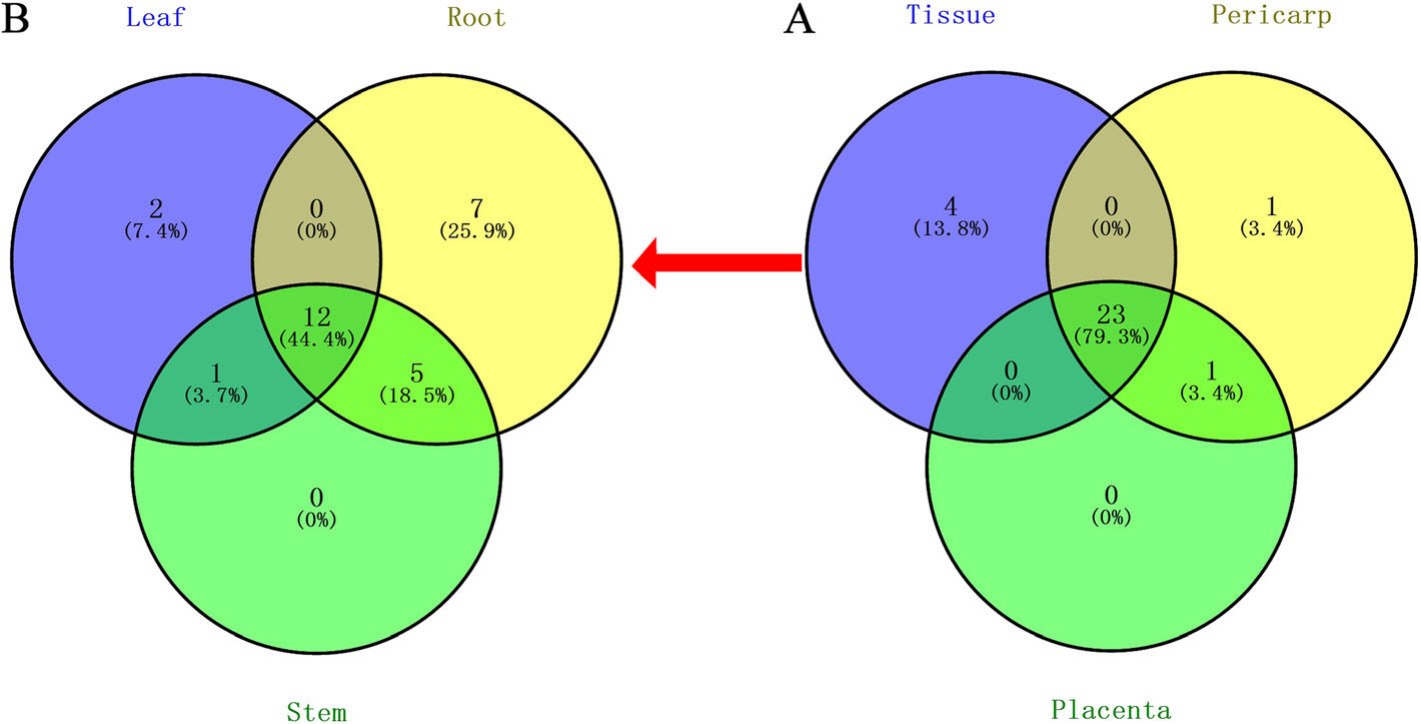
Number of expressed *CaDnaJ*s in different tissues in CM334. The interaction of pericarp, placenta and different vegetative tissues (A) and the number of expressed *CaDnaJ*s in different (roots, stem and leaves) (B) are demonstrated in the figure

Twenty-nine *CaDnaJ* genes in different tissues with RPKM value greater than 5.0 were selected for further analysis (Supplement Table S2). As it shown in Fig. 4, few CaDnaJs were specifically expressed in the tested tissues, and most *CaDnaJs* were expressed in two or more tissues. Data analysis showed that the number of specifically expressed *CaDnaJs* in vegetative tissue (leaf, root, and stem) was higher than that in reproductive tissue (pericarp and placenta) (Table 2). Additionally, more CaDnaJ genes were preferentially expressed in pericarp compared to the other tissues. Leaf and placenta share the same quantity of the preferentially expressed genes (Table 2).

**Table 2:** The number of expressed, preferentially expressed, and specifically expressed *CaDnaJ* genes in different tissues in pepper

Type	Leaf	Root	Stem	Pericarp	Placenta
Expressed CaDnaJs	15	24	18	25	24
Preferentially expressed CaDnaJs	5	6	3	10	5
Specifically expressed CaDnaJs	2	7	0	1	0

### Expression patterns of CaDnaJ genes in placenta

For transcriptome analyses of fruit, pericarp and placenta were harvested from CM334 (pungent) plants at the stages of 6 days post-anthesis (DPA), 16 DPA, 25 DPA, mature green (MG), breaker (B), 5 days post-breaker (B5), and B10. And in ECW30R (non-pungent) plants, placenta was harvested at 6 DPA, 13 DPA, 20 DPA, MG, B, B5, and B10 stages [16]. To reveal whether the expression of DnaJ is involved in the synthesis of capsaicin, CM334 and ECW30R were used to investigate expression patterns of *CaDnaJ* genes in placenta. Comparison analysis of RNA-seq data revealed that eight genes out of expressed 29 CaDnaJs (CaDnaJ25, 47 and 56 from sub-family III, CaDnaJ10, 40 and 74 from sub-family IV, CaDnaJ70 from sub-family V and CaDnaJ46 from sub-family VII) exhibited distinct expression profiles in CM334 and ECW (Supplemental Figure S1).

For CM334, the expression of CaDnaJ10, 25 and 40 increased gradually before the stage of MG and then remained high expression, while CaDnaJ47 and 70 remained stable during the development of placenta. In particular, the CaDnaJ46 was barely expressed before the stage of B, and then dramatically increased in the later period. The expression of CaDnaJ56 were dramatically down-regulated in early stage and remained stable (Supplemental Figure S1).

In ECW, the expression of CaDnaJ25, 47 and 74 increased gradually at the early stage of placenta development, and then remain low expression. The expression of CaDnaJ40, 46 and 74 in ECW increased gradually with the development of the placenta. On the contrary, expressions of CaDnaJ70 were down-regulated with the development of the placenta and were scarcely expressed in B10. Furthermore, expressions of CaDnaJ10 and 56 remained nearly stable during the whole-placenta develop stage (Supplemental Figure S1).

The expressions of CaDnaJ47, 56 and 70 in CM334 were higher than those in ECW during whole development of placenta, while the CaDnaJ10 and 25 showed higher expressions only after the stage of MG. On the contrary, the expressions of CaDnaJ40 and 46 were lower in CM334 than those in ECW during whole development of placenta.

**Fig. 5:**
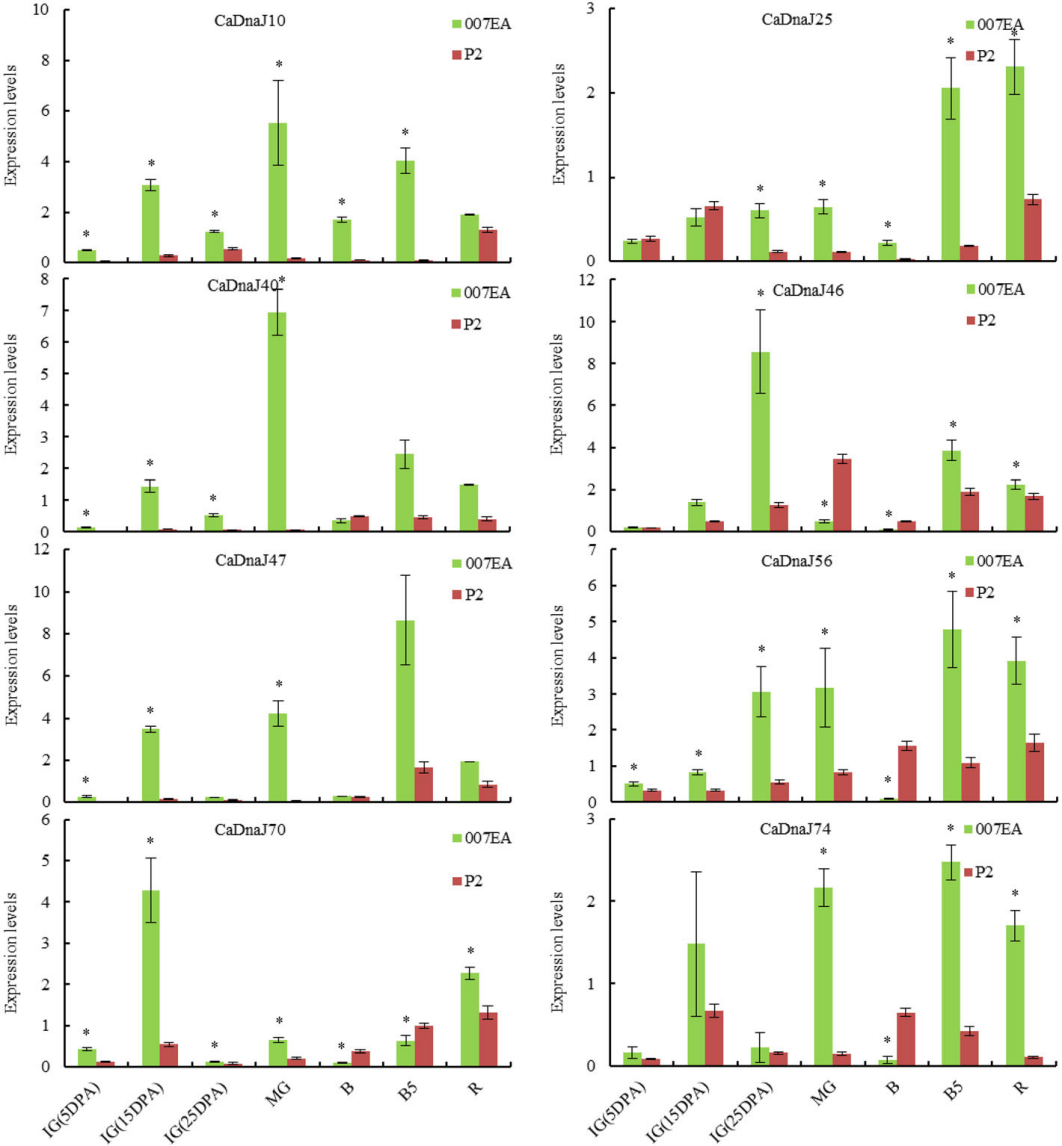
The relative expressions of 8 *CaDnaJ* genes in placenta from 007EA (pungent) and P2 (non-pungent) on 7 different development stage. The expression levels of these *CaDnaJ*s were tested using qRT-PCR. IG (5DPA), immature green fruit (5 days post-anthesis); IG (15DPA), immature green fruit (15 days post-anthesis); IG (25DPA), immature green fruit (25 days post-anthesis); MG, mature green fruit; B, breaker fruits; B5, breaker+ 5 fruits; R, mature red fruits. qRT-qPCR was performed in three biological replicates of different development stages of fruit samples. Bars represent the standard deviation (±SD) calculated for three biological replicates. Statistically significant differences are indicated p < 0.05 by star (*) (Student’s t-test)

In order to verify the expressions of eight CaDnaJ genes in the non-pungent and pungent cultivar for further, two accessions, the pepper P2 (non-pungent) and 007EA (pungent) at different development stages of placenta, were used to test and confirm their expressions by qRT-PCR. As shown in Fig. 5, all eight CaDnaJ genes showed distinct expression profiles. In P2 and 007EA, the expression of 6 genes (CaDnaJ10, 25, 56, 70, 47 and 74) out of 8 CaDnaJs were mostly consistent with data from the RNA-seq in CM334 and ECW. Detail analysis revealed that the expression of CaDnaJ10, 25, 40, and 47 were up-regulated at different placenta development stages. The expression levels of CaDnaJ56, CaDnaJ70 and CaDnaJ74 were down-regulated at the stage of B, but were up-regulated at the other six stages. On the contrary, in P2 and 007EA, the expression of CaDnaJ40 showed opposed results from the CM334 and ECW.

### The CaDnaJs co-expressed with genes involved in capsaicinoids synthesis during placenta development

The specific regulations of *CaDnaJs* at placenta development stages prompted us to examine whether the *CaDnaJs* co-expressed with genes involved in capsaicinoids synthesis. Previously, nine capsaicinoid-biosynthetic genes (CBGs) have been identified [16]. In this study, expression patterns of these nine genes during placenta development were performed based RNA-seq (Supplemental Figure S2). The result showed that expression levels of all genes related to capsaicinoids synthesis in immature green fruit (5DPA, 15DPA and 25DPA) were higher than that in mature red fruit (break, break+ 5 and red fruit). Among these nine genes, expressions of three genes (Pal1, BCAT and C4H) in 007EA (high pungent) were lower than that in P2 (non-pungent), which does not relate to the capsaicin accumulation at placenta developmental stage. For *KAS* and *FatA* genes, we found that expression levels of these two genes were higher in P2 than that in 007EA at 15DPA; however, they were lower in fruit at 25DPA. These two genes (KAS and FatA) were potentially co-expressed with *CaDnaJs* at immature fruit (25 DPA) (Supplemental Figure S1). Expression of the remaining four genes (ACL, COMT, AMT and CS) showed higher in 007EA than that in P2, which was consistent with expression of *CaDnaJs* (Supplemental Figure S1). Overall, these nine genes in the pepper genome by examining their tissue-specific expression profiles indicated that six genes appear to be co-expressed with *CaDnaJs* during placenta development.

### Expression patterns of CaDnaJ genes in response to different kinds of stress condition

**Fig. 6:**
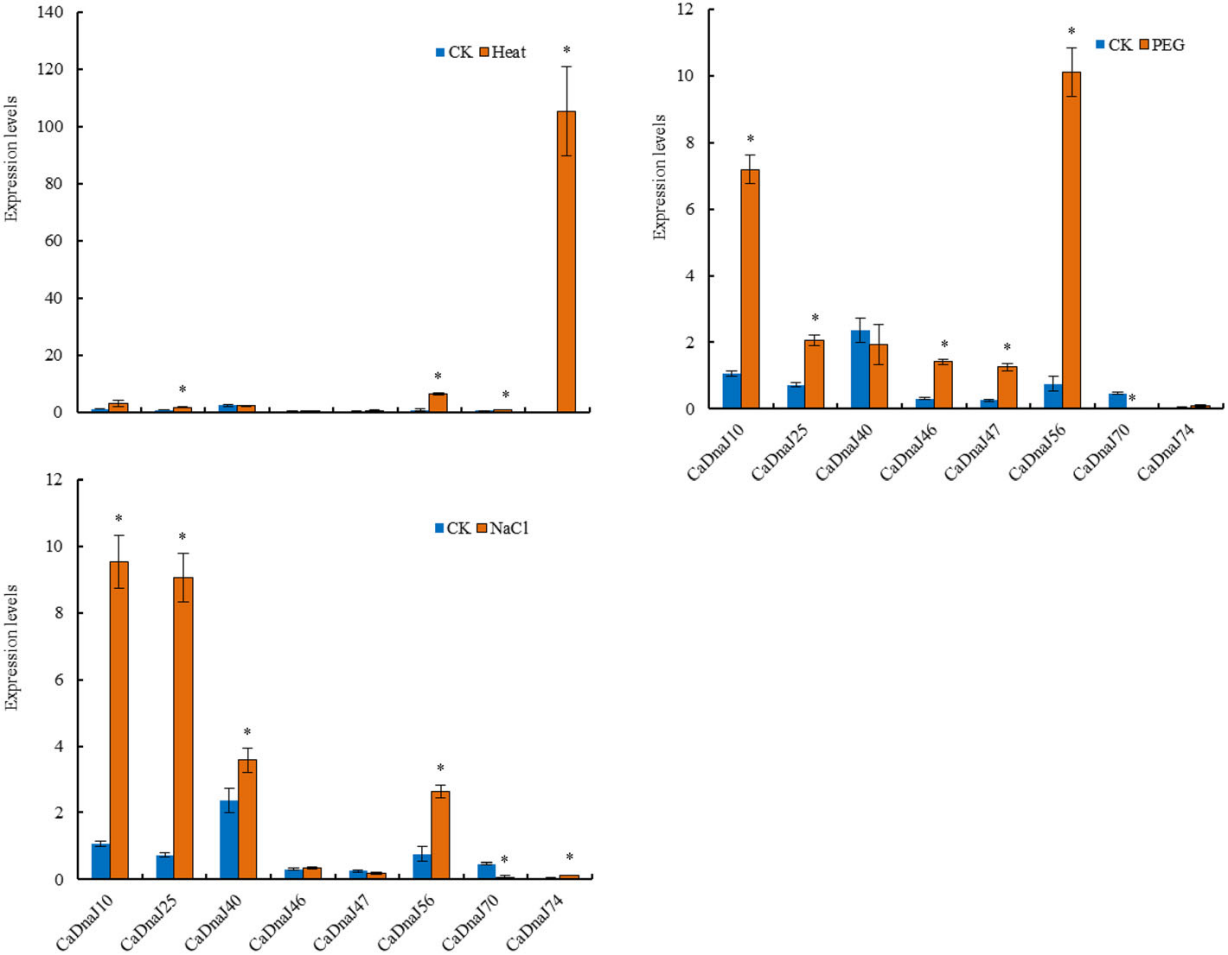
The relative expressions of 8 *CaDnaJ* genes in the leaves of 007EA (pungent) at the stage of 6–8 true leaves in response to abiotic stresses, including heat, PEG and Nacl. Each treatment was conducted with three biological replicates, and samples from five plants were collected for each replicate. qRT-qPCR was performed in three biological replicates of the samples. Bars represent the standard deviation (±SD) calculated for three biological replicates. Statistically significant differences are indicated p < 0.05 by star (*) (Student’s t-test)

Abiotic stresses like salinity, heat, and polyethylene glycol (PEG) adversely affect the growth and physiological processes of plants [28–39]. In this study, expression patterns of eight CaDnaJ genes related to biosynthesis of compounds related with pungency were further investigated under various abiotic stresses to explore their potential roles in stress tolerance. With the help of qPCR, the response of 8 CaDnaJs to stress conditions, including heat, PEG and salt, was analyzed. The results showed that these eight genes were regulated by heat, PEG and salt, respectively (Fig. 6). The expression of CaDnaJ74 was greatly up-regulated under high temperature stress, implying that CaDnaJ74 may be involved in the process of plant response to high-temperature stress. Under drought stress, the expression of eight CaDnaJ genes presented noticeable changes. This result showed that expressions of 5 genes (CaDnaJ10, 25, 46, 47 and 56) out of 8 *CaDnaJs* were significantly up-regulated. By contrast, CaDnaJ70 was down-regulated. The results imply that these CaDnaJs might participate in drought stress responses. Compared to expression levels under drought stress, only CaDnaJ70 and 74 were down-regulated during salt stress.

### Expression patterns of CaDnaJ genes under hormone treatment

**Fig. 7:**
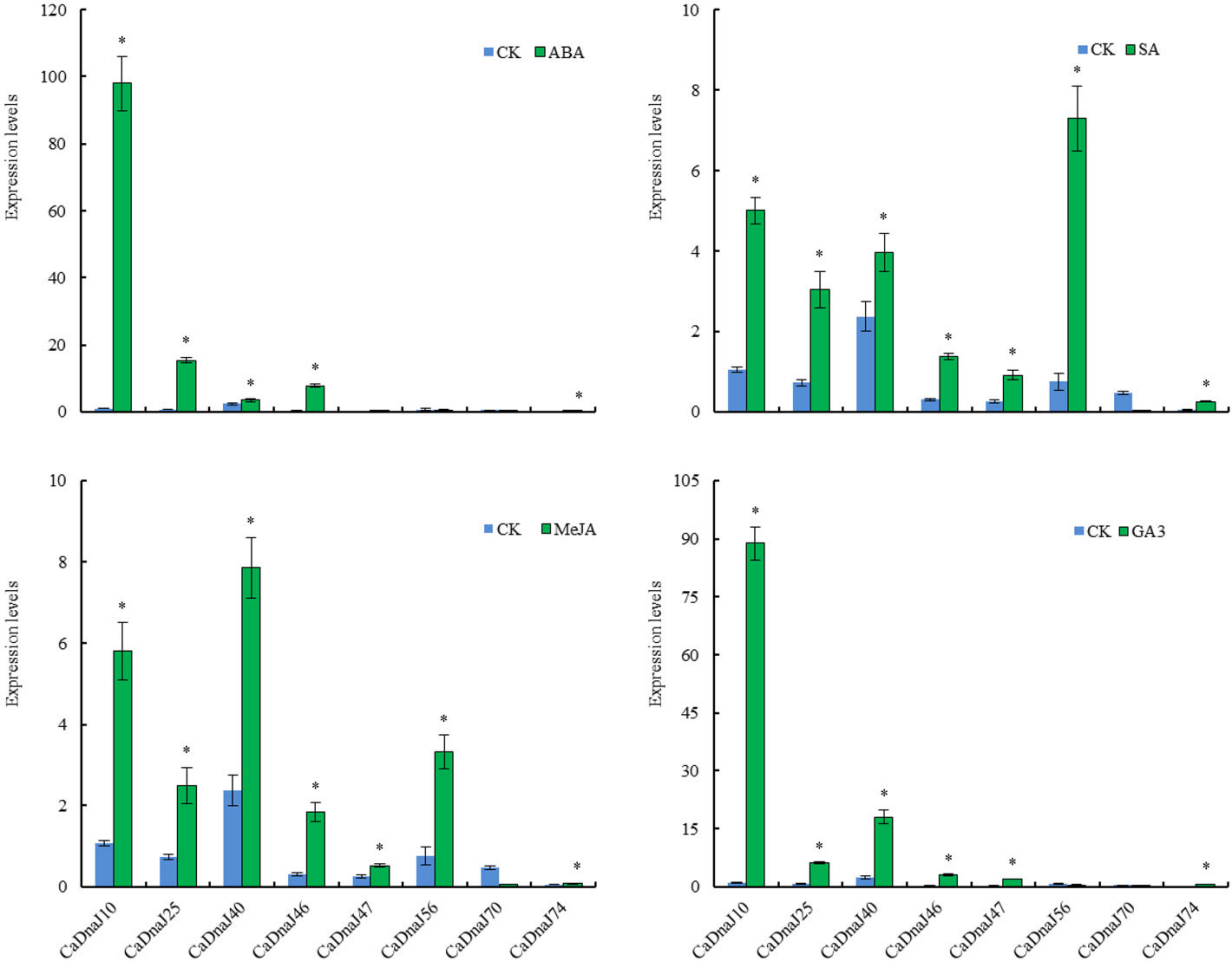
The relative expressions of 8 *CaDnaJ* genes in the leaves of 007EA (pungent) at the stage of 6–8 true leaves in response to plant hormones, including ABA, GA3, MeJA and SA. Each treatment was conducted with three biological replicates, and samples from five plants were collected for each replicate. qRT-qPCR was performed in three biological replicates of of the samples. Bars represent the standard deviation (±SD) calculated for three biological replicates. Statistically significant differences are indicated p < 0.05 by star (*) (Student’s t-test)

Plant hormones regulate multiple aspects of plant growth and development and mediate environmental responses to ensure a successful life cycle [27, 40–47]. In this study, in order to investigate the roles of the CaDnaJ genes in response to hormone treatments, the seedlings at the age of 6–8 true leaves were subjected to abscisic acid (ABA), GA_3_, methyl jasmonate (MeJA) and salicylic acid (SA) treatments (Fig. 7). For ABA treatment, four genes (CaDnaJ10, 25, 40 and 46) were remarkably up-regulated (Fig. 7). For GA_3_ treatment, CaDnaJ10, 25, 40, 46, 47 and 74 also were up-regulation except for CaDnaJ56 and 70 genes. Both MeJA and SA treatments induced up-regulation of 7 genes (CaDnaJ10, 25, 40, 46, 47, 56 and 74). On the contrary, CaDnaJ70 was down-regulated in all tested hormone treatments (Fig. 7). Our results suggested that these eight CaDnaJ genes were induced by these hormone treatments.

## Discussion

Because of its rich capsaicin, nutrients and pigments in the fruits, pepper has become the world’s second most popular vegetable after tomato [48, 49]. Recently, the DnaJ proteins, ubiquitously participate in various plant growth and development processes, have been identified in pepper [23]. In the present study, the comprehensive expression profiles of entire 76 pepper DnaJ genes were analyzed in detail. Our result showed that about 38% (29) of CaDnaJs were expressed in at least one tissues (Supplement Table S2), indicating the potentially critical role of CaDnaJs in plant growth and development in pepper. Further analysis of these 29 CaDnaJs revealed that 5, 6 and 3 CaDnaJs were highly expresssed in leaf, root and stem, respectively, suggesting fundamental function in pepper vegetative growth stage (Supplement Table S2; Table 2). Additionally, we also found that 10 and 5 CaDnaJs were highly expresssed in pericarp and placenta, respectively, indicating a conservation role of these genes in pepper reproductive growth stage (Supplement Table S2; Table 2). Moreover, expression of some CaDnaJ genes was tissue-specific. For example, CaDnaJ27 and 66 were specifically expressed in leaf. Seven genes, CaDnaJ 07, 10, 13, 44, 45, 46 and 74, were specifically expressed in root. The CaDnaJ 04 was specifically expressed in pericarp. These results revealed that these genes may be associated with the regulation of growth and development of these specific tissues.

The pungent properties of peppers are attributed to a group of compounds called capsaicinoids, unique alkaloids that accumulate only in pepper fruit. These compounds are becoming increasingly important in food, cosmetic and pharmaceutical industries. The capsaicinoids are found generally in the white part inside the pepper, which were called placenta. Currently, the capsaicinoids biosynthetic pathways, including the key CBGs and the related transcription factors (TFs), such as *Erf*, *Jerf* and *CaMYB31*, have been largely elucidated [50, 51]. To analyze the relationship between expression profiles of DnaJ genes and capsaicinoids synthesis, nine genes involved in capsaicinoids synthesis (*Pal1, BCAT, C4H, KAS, ACL, COMT, FatA, AMT* and *CS*) were selected in the pungent (007EA) and non-pungent peppers (P2) in this study [16]. Among them, we found that six genes (*KAS, FatA, ACL, COMT, AMT* and *CS*) genes were higher in P2 than that in 007EA at IG (5DPA, 15DPA and 25DPA), which was consistent with expressions of eight *CaDnaJs* during placenta development. Co-expression patterns between *CaDnaJs* and the genes related to capsaicinoids synthesis were found, which implied that these *CaDnaJs* genes might participate in the regulation of capsaicinoids synthesis in pepper.

Usually, understanding of how and where each gene functions demands detailed knowledge of spatial-temporal gene expression patterns [43, 52, 53] and in response to various abiotic stresses [54–57]. The biosynthesis and accumulation of capsaicinoids in chili pepper fruits were affected by various internal and environmental factors [58], including plant hormones like indoleacetic acid (IAA), jasmonic acid (JA), salicylic acid (SA), and gibberellic acid (GA), and stress conditions such as temperature, light, wounding and drought. Moreover, the environment effects on fruit yield and capsaicinoids production might vary depending on the chili genotypes. Previous studies in seven hot pepper hybrid cultivars and two commercial cultivars confirmed that a large proportion of variation on capsaicinoid yield (67.7%) was contributed from environment condition while variations due to genotype were only 42.4% [50, 59]. On the contrary, Tripodi et al. [60] reported that the environment accounted for less than 0.5% for capsaicinoids in 14 hot pepper accessions cultivated in two different pedoclimatic locations.

In our study, eight *CaDnaJ* genes (CaDnaJ10, 25, 40, 46, 47, 56, 70 and 74) with similar expression pattern to the CBGs in placenta of CM334 (pungent) and ECW (non-pungent) were selected to investigate their potential roles in pepper responses to various abiotic stresses and hormones. The expression levels of these eight *CaDnaJ* genes under heat, drought and salt were determined by qRT-PCR (Fig. 6). Some researchers had suggested that capsaicin production was improved under limited water supply in the low and medium pungency cultivars [61]. In contrast, the reduction or no changes of capsaicinoid production under drought condition also was found [59, 62]. Our data showed that drought condition had greater effect on the expression of these eight genes than the other two abiotic stresses. The expressions of 6 genes out of 8 *CaDnaJs* were significantly increased in response to drought stress. The expression levels of only two genes (CaDnaJ40 and CaDnaJ70) were slightly decreased. The result supported previous study that the synthesis of capsaicinoids was sensitive to water stress.

In previous studies, it was reported that SA, IAA and GA_3_ treatment promoted the expression of three capsaicinoid structural genes, *CaMYB31*, *Kas*, and *pAmt*, while JA treatment caused a significant decrease in their expression, except at 3 h of exposure [50]. In our study, the qRT-PCR analysis showed that the expression of these eight CaDanJ genes was up-regulated or down-regulated after 4 different hormone treatments including ABA, GA_3_, MeJA and SA (Fig. 7). However, we did not observe an antagonistic effect of MeJA on the expression of these eight CaDanJ genes compared with the ABA, GA_3_ and SA. This phenomenon supported the fact that the level of pungency in hot pepper was either positively or negatively regulated by different plant hormones. The concentration of the plant hormone and exposure time highly influence the pungency level.

## Conclusions

In our study, a genome-wide identification and expression analysis of the 76 *CaDnaJ*s were performed at the whole genome level. Eight *DnaJ* genes involved in the regulation of capsaicinoids synthesis were confirmed by the co-expression patterns of these CaDnaJ genes and CBGs. Furthermore, these eight genes were induced by various abiotic stresses (heat, drought and salt) and plant hormones (ABA, GA3, MeJA and SA), which revealed the influence of environmental factors in the biosynthesis of compounds related with pungency. These results provide new information that may facilitate the further functional analysis of the pepper *CaDnaJ*s.

## Methods

### Data sources

The genomic sequences of pepper downloaded from the Pepper Genome Database (PGD, http://peppergenome.snu.ac.kr/) [16].

### Phylogenetics analysis

The full amino acid sequences of 76 CaDnaJ members from *Capsicum annuum* CM334 were aligned by ClustalX program. The phylogenetic tree was built using MEGA5.10 software [63]. The Neighbor-joining method, pair wise deletion and a Poisson model were used with a bootstrap (1000 replicates) test of phylogeny. The pepper CaDnaJ genes were assigned to different groups based on the multiple sequence alignment and the classification of DnaJ genes in the other four species.

### Expression analysis of pepper DnaJ in various tissues

RNA-seq data, which have been revealed by previous researchers [16], were used to investigate expression patterns of *CaDnaJ* genes in the roots, stems, and leaves from 6-week-old CM334 plants.

RPKM (Reads Per Kilo bases per Million mapped Reads) values of *CaDnaJ* genes were log2- transformed [64]. Based on the expression levels and patterns, the genes were defined as expressed, specifically expressed and preferentially expressed according to the classification criteria of Cheng et al. [65].

### Plant growth and stress treatments

Two inbred lines provided by our lab, P2 (non-pungent) and 007EA (pungent), were cultivated in the greenhouse at the Zhejiang Academy of Agricultural Sciences. The placenta were harvested from P2 and 007EA plants to investigate expression patterns of *CaDnaJ* genes at different development stage, including immature green fruit (5DPA, 15DPA and 25DPA), mature green fruit, breaker fruits, breaker+ 5 fruits and mature red fruits.

For hormone treatments, seeds for hot pepper 007EA were sterilized for 5 min using 10% hypochlorous acid solution. Germinated seeds were sown in plastic pots with substrate (peat:perlite-3:1 by volume) in a growth chamber, under 16-h photoperiod at 26 °C/19 °C till reaching the age of 6–8 true leaves. Subsequently, the seedlings were sprayed with 100 μM Methyl jasmonate (MeJA), 100 μM Gibberellin (GA_3_), 100 μM abscisic acid (ABA), 1000 μM salicylic acid (SA) solutions, and leaves were collected 4 h later.

The seedlings of hot pepper var. 007EA were root irrigated 200 mL of 50 mM NaCl solution to the substrate per plant for salt stress, and 200 mL of 18% PEG for drought stress treatments. For heat stress treatments, the plants were treated with 42 °C by placing in a light incubator and plants grown at 25 °C were used as the control group. The leaves of all treatments were sampled for analysis 4 h later.

Three biological replicates of samples of hormone treatments (MeJA, GA_3_, ABA and SA) and abiotic stress treatments (42 °C and Nacl) were collected from pepper 007EA planted in the greenhouse of Zhejiang Academy of Agricultural Sciences (Hangzhou, China). Each biological replicate contains five individuals. We collected five leaves from each individual and mixed them together as one biological replicate. The samples were collected and immediately frozen with liquid nitrogen for total RNA extraction.

### Total RNA extraction, reverse transcriptions and qRT-PCR analysis

Total RNA extraction and reverse transcription were performed using Total RNA kit and FastQuant RT Kit (Tiangen Biotech, Beijing, China) according to the manufacturers’ protocol.

The ABI StepOne Plus Real-time PCR system was used for quantitative RT-PCR analysis. The procedure was done according to the instructions in the SYBR qPCR Master Mix (Vazyme Biotech co., Ltd., Nanjing, China). Cycling conditions were 94 °C for 5 min, followed by 33 cycles of 94 °C for 30 s, 55 °C for 30s and extension at 72 °C for 30s. The *UBI* gene was used as an internal control [66]. Gene-specific primers used for amplification were listed in Supplement Table S1. The expression values of CaDnaJ genes were calculated by the 2^-△ct^ method.

### Statistical analysis

All experiments were done with three biological repeats, and five plants per treatment were collected for analysis. All data were statistically analyzed with SPSS software (https://www.ibm.com/analytics/spss-statistics-software) using student’s t-test at the 0.05 level of significance.

## Supplementary information

**Additional file 1 Table S1** Primers used for qRT-PCR in this study. **Table S2** Fragments per kilobase of exon model per million mapped (FPKM) values of all the CaDnaJs in different pepper tissues. **Supplemental Fig. S1** The relative expressions of 8 *CaDnaJ* genes in placenta from CM334 (pungent) and ECW (non-pungent) based on RNA-seq data. 6DPA, 6 days post-anthesis; 16 DPA, 16 days post-anthesis; 25 DPA, 25 days post-anthesis; MG, mature green; B5, 5 days post-breaker; B10, 10 days post-breaker. **Supplemental Fig. S2** The RNA-Seq data of nine capsaicinoid-biosynthetic genes during the different stage of placenta from CM334 (pungent) and ECW (non-pungent). IG (6DPA), immature green fruit (5 days post-anthesis); IG (16DPA), immature green fruit (16 days post-anthesis); IG (25DPA), immature green fruit (25 days post-anthesis); MG, mature green fruit; B, breaker fruits; B5, breaker+ 5 fruits; R, mature red fruits.

## Data Availability

All data used during the current study are included in this published article or are available from the corresponding author on reasonable request.

## Abbreviations

ABA: Abscisic acid
B: Breaker
B5: 5 days post-breaker
CBGs: Capsaicinoid-biosynthetic genes
DPA: Days post-anthesis
GA_3_: Gibberellin
IAA: Indoleacetic acid
JA: Jasmonic acid
MeJA: Methyl jasmonate
MG: Mature green
RPKM: Reads Per Kilo bases per Million mapped Reads
SA: Salicylic acid
TFs: Transcription factors
