# Supplementary figures and images for: Global analysis of expression profile of members of DnaJ gene families involved in capsaicinoids synthesis in pepper (Capsicum annuum L)
Source: BMC Plant Biol. 2020 Jul 9;20:326. doi: 10.1186/s12870-020-02476-3 (PMC7350186; doi:10.1186/s12870-020-02476-3)

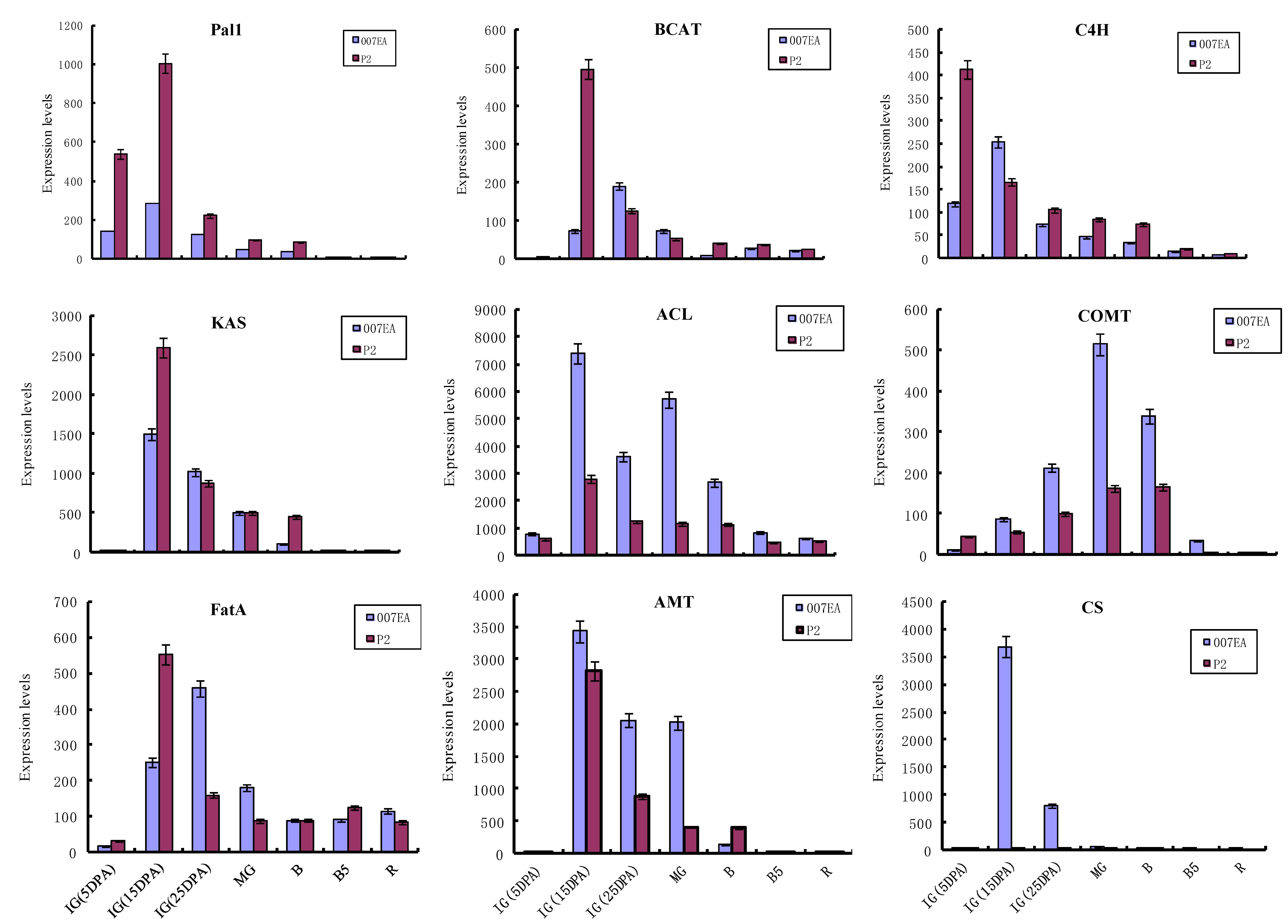

Supplement: Supplementary file 1 — Additional file 1 Table S1 Primers used for qRT-PCR in this study. Table S2 Fragments per kilobase of exon model per million mapped (FPKM) values of all the CaDnaJs in different pepper tissues. Supplemental Fig. S1 The relative expressions of 8 CaDnaJ genes in placenta from CM334 (pungent) and ECW (non-pungent) based on RNA-seq data. 6DPA, 6 days post-anthesis; 16 DPA, 16 days post-anthesis; 25 DPA, 25 days post-anthesis; MG, mature green; B5, 5 days post-breaker; B10, 10 days post-breaker. Supplemental Fig. S2 The RNA-Seq data of nine capsaicinoid-biosynthetic genes during the different stage of placenta from CM334 (pungent) and ECW (non-pungent). IG (6DPA), immature green fruit (5 days post-anthesis); IG (16DPA), immature green fruit (16 days post-anthesis); IG (25DPA), immature green fruit (25 days post-anthesis); MG, mature green fruit; B, breaker fruits; B5, breaker+ 5 fruits; R, mature red fruits. [file 12870_2020_2476_MOESM1_ESM.zip › Supplemental Figure S2.jpg]

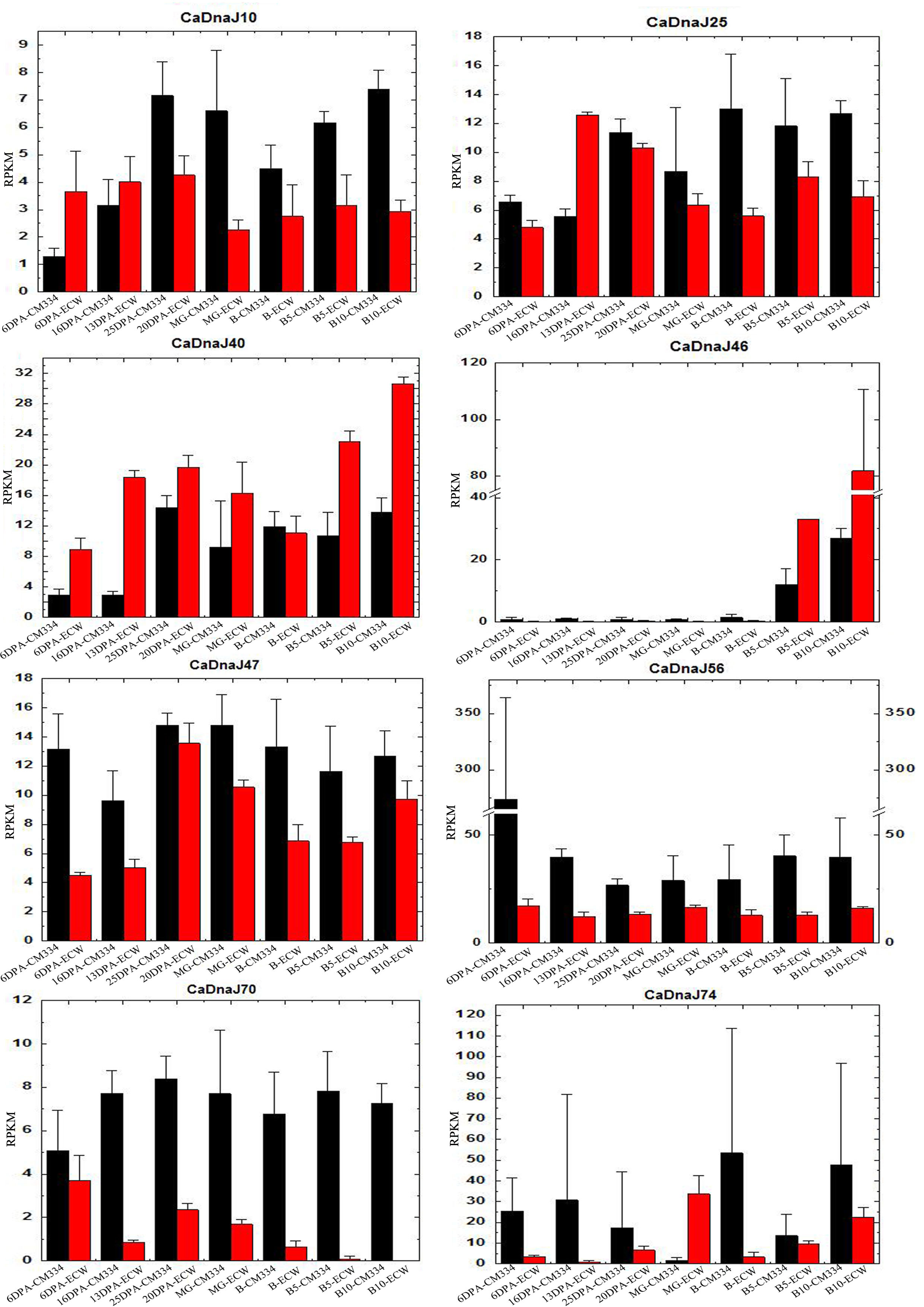

Supplement: Supplementary file 1 — Additional file 1 Table S1 Primers used for qRT-PCR in this study. Table S2 Fragments per kilobase of exon model per million mapped (FPKM) values of all the CaDnaJs in different pepper tissues. Supplemental Fig. S1 The relative expressions of 8 CaDnaJ genes in placenta from CM334 (pungent) and ECW (non-pungent) based on RNA-seq data. 6DPA, 6 days post-anthesis; 16 DPA, 16 days post-anthesis; 25 DPA, 25 days post-anthesis; MG, mature green; B5, 5 days post-breaker; B10, 10 days post-breaker. Supplemental Fig. S2 The RNA-Seq data of nine capsaicinoid-biosynthetic genes during the different stage of placenta from CM334 (pungent) and ECW (non-pungent). IG (6DPA), immature green fruit (5 days post-anthesis); IG (16DPA), immature green fruit (16 days post-anthesis); IG (25DPA), immature green fruit (25 days post-anthesis); MG, mature green fruit; B, breaker fruits; B5, breaker+ 5 fruits; R, mature red fruits. [file 12870_2020_2476_MOESM1_ESM.zip › Supplemental Figure S1.jpg]
